# Synovial fibroblast responses to different types of injury resulting in cartilage repair or osteoarthritis

**DOI:** 10.1016/j.joca.2025.12.023

**Published:** 2026-01-03

**Authors:** Fraser L. Collins, Alexander J. Knights, Tristan Maerz, Anke J. Roelofs, Cosimo De Bari

**Affiliations:** a Rheumatology Research Group, Institute of Genetics and Cancer, University of Edinburgh, UK; b Department of Orthopaedic Surgery, University of Michigan, Ann Arbor, MI, USA; c Department of Orthopaedic Surgery, Washington University, St Louis, MO, USA; d Center of Regenerative Medicine, Washington University, St Louis, MO, USA; e Institute for Biomechanics, Department of Health Sciences and Technology, ETH Zürich, Zürich, Switzerland; f Departments of Biomedical Engineering and Internal Medicine (Division of Rheumatology), University of Michigan, Ann Arbor, MI, USA

**Keywords:** Synovial fibroblast, Progenitor, Injury, Osteoarthritis

## Abstract

**Objective:**

Single-cell RNA-sequencing (scRNA-seq) has advanced our understanding of the heterogeneity of synovial fibroblasts and their roles in tissue repair and osteoarthritis. Here, we compared the fibroblast responses to different types and duration of joint injury in mice.

**Design:**

Published scRNA-seq data of synovial fibroblasts from a model of joint surface injury (JSI, day 6) and two post-traumatic osteoarthritis models, destabilisation of the medial meniscus (DMM, day 7 and 2 months) and anterior cruciate ligament rupture (ACLR, day 7 and day 28), were integrated and analysed using clustering, DEG, gene-set enrichment, regulon, pseudotime trajectory and cell-cycle analyses.

**Results:**

The response to JSI was comparatively dominated by lining progenitor and fibroblast-like synoviocyte (FLS) expansion, while DMM and ACLR induced transient expansion of perturbed-state fibroblasts (PSF) containing a myofibroblast subset, followed by FLS expansion over time. Early injury responses were underpinned by proliferating cells that directly supplied new PSF and FLS. Molecular pathway analysis revealed rapid and sustained injury-induced transcriptomic shifts in both lining and sublining fibroblasts, with post-traumatic OA models in particular showing marked upregulation of matrix-related genes indicative of myofibroblast activity and tissue remodeling. Activity of regulons associated with the PSF response, including Hes6, Hif1α, Zfp354c, Runx1 and Foxp1, showed model and stage-dependent upregulation. Finally, co-expression of lineage-specifying transcription factors *Sox9*, *Runx2* and *Scx*, indicative of multilineage potential, was a common feature of PSF in all injury models.

**Conclusions:**

This study uncovers similarities and differences in synovial fibroblast responses between injury models, guiding future research on joint repair and osteoarthritis.

## Introduction

Joint injury, a risk factor for osteoarthritis [1], triggers proliferation and expansion of fibroblasts in synovium, the thin membrane that lines the inner surface of synovial joints, leading to synovial hyperplasia. This can be associated with inflammation and develop into fibrosis in osteoarthritis [2].

Fibroblast is an umbrella term describing diverse stromal cells that form and remodel the extracellular matrix, support local cellular niches, regulate immune system responses within tissues, and orchestrate repair after injury [3]. Single-cell RNA-sequencing (scRNA-seq) has greatly advanced our understanding of diverse fibroblast cell states and fibroblast-driven disease mechanisms [4–6]. Buechler *et al*. investigated fibroblast transcriptional states across multiple tissues and identified conserved fibroblast subsets, including “universal fibroblasts” marked by expression of *Pi16* and *Dpp4* and perturbed-state fibroblast subsets across various diseases [4]. In inflammatory arthritis, scRNA-seq analysis identified pathogenic synovial fibroblasts that drive disease [6–8], and clinical trials targeting fibroblasts for the treatment of rheumatoid arthritis are emerging [9–11].

Recent studies employing scRNA-seq in mouse models of joint injury and post-traumatic osteoarthritis have revealed synovial fibroblast responses to injury and OA at unprecedented resolution [12–14]. These cells can broadly be divided into specialised fibroblast-like synoviocytes (FLS) located in the synovial lining, which provide joint lubrication through secretion of lubricin (encoded by *Prg4*) and hyaluronan, and a heterogeneous pool of fibroblasts in the sublining, which provide structural support and immune regulation [4,5]. In addition, both lining and sublining contain fibroblasts that show functional properties of stem/progenitor cells after injury [15].

Fibroblasts with stem/progenitor cell characteristics in synovial lining, here referred to as lining progenitor (LinP) cells, share with the FLS an ontogenetic derivation from the *Gdf5*-expressing joint interzone and the expression of *Prg4* [12,16]. Following acute cartilage injury, LinP cells proliferate to supply new FLS [12]. In addition, *Prg4*-traced progeny can populate the cartilage defect [17] and contribute to cartilage repair [18]. *Prg4*-traced progeny can also extensively invade the sublining tissue while losing Prg4 expression [17], and in the destabilisation of the medial meniscus (DMM) model of OA they were shown to adopt a myofibroblast-like phenotype [13] and differentiate into chondrocytes leading to osteophyte formation [19]. Fibroblasts with stem/progenitor cell characteristics in synovial sublining, here referred to as sublining progenitor (SubP) cells, are postulated to be equivalent to the *Dpp4*-expressing universal fibroblasts identified by Buechler *et al*. [4] and an analogous population of adipose stromal progenitors [20], and were bioinformatically predicted to give rise to mature cell types [12–14]. Experimental evidence to support this comes from a recent study showing that *Dpp4*-traced progeny supply adipocytes to the infrapatellar fat pad during postnatal growth [13]. In addition, *Dpp4*-traced cells proliferated and expanded throughout synovium in response to DMM and gave rise to myofibroblast-like cells in sublining and *Prg4*-expressing cells in lining [13].

Various mouse models of joint injury and OA are used in preclinical research [12–14], each with respective advantages and limitations [21,22], and it is unclear whether the synovial fibroblast response differs between models. Here, we integrated scRNA-seq datasets from three mouse models of joint injury to comparatively assess synovial fibroblast transcriptomes, hypothesising that the dynamic changes in their cellular states depend on the type of injury. We find commonalities and define differences in the injury-induced synovial fibroblast populations across models, furthering our understanding of reparative mechanisms and OA progression. Our study also provides a knowledge base that will guide targeted design and interpretation of studies employing mouse models of injury.

## Methods

Synovial scRNA-seq datasets were downloaded from the Gene Expression Omnibus (GEO) database (Supplementary Table 1). All datasets were derived from multiple mice (2–20 per group), as detailed in Supplementary Table 2. Data analysis was performed using the Seurat R package (version 5.0.2) [23]. Poor-quality data was removed by excluding cells with fewer than 200 genes, more than 4000 (control samples) or 6000 genes (post-injury samples), or greater than 5% mitochondrial genes. In addition, cells negative for fibroblast markers *Pdpn* or *Pdgfra* and expressing either the hematopoietic cell markers *Ptprc* or *Fcer1g*, or the endothelial marker *Pecam1,* were computationally removed. Datasets were integrated by reciprocal PCA, with 2000 integration anchor features that were repeatedly variable across datasets, and gene expression measurements were normalised by total expression, multiplied by a scale factor (10,000) and log-transformed. Cell cycle regression was performed using the Seurat CellCycleScoring function. Fifty principal components were used for UMAP and clustering analysis (original Louvain algorithm). Clustering resolution, determined using the R package Clustree [24] and differentially-expressed gene (DEG) analysis, was set at 0.3. To identify DEGs, genes detected in at least 25% of cells either within or outside the cluster of interest and demonstrating a minimum log2-fold difference of 0.25 were analysed for differential expression using the non-parametric Wilcoxon rank sum test with Bonferroni correction using all features in the dataset, with significance level set at P < 0.05. Comparative gene expression heatmaps were generated using the Seurat ‘AggregateExpression’ function to create a pseudobulk dataset. A pseudocount of +1 was added to each value, to account for samples with no activity, and log2-fold-change calculated relative to each model’s respective steady-state control. Visualisation of gene co-expression, based on gene-weighted density estimation, was performed with the R package Nebulosa [25]. Reactome analysis was performed using the ReactomeGSA package [4], GSVA Hallmark pathway analysis with the GSVA package [5], regulon analysis using the SCENIC package [8], trajectory analysis using the Monocle3 package [10], and cell cycle analysis using the Tricycle package [11], as detailed in Supplementary Methods. Statistical analysis of regulon activity was performed by multivariate analysis of variance (MANOVA) followed by pairwise comparisons with Benjamini-Hochberg correction for multiple comparisons. Data for each injury model were normalized to their respective steady-state control and log2-fold-change calculated. Statistical analysis of relative abundance was performed by Pearson’s Chi-squared test, followed by within-injury-model Pearson’s Chi-squared pairwise comparisons with Benjamini-Hochberg correction for multiple comparisons.

## Results

### Harmonisation of synovial fibroblast populations across mouse models of joint injury

**Fig. 1:**
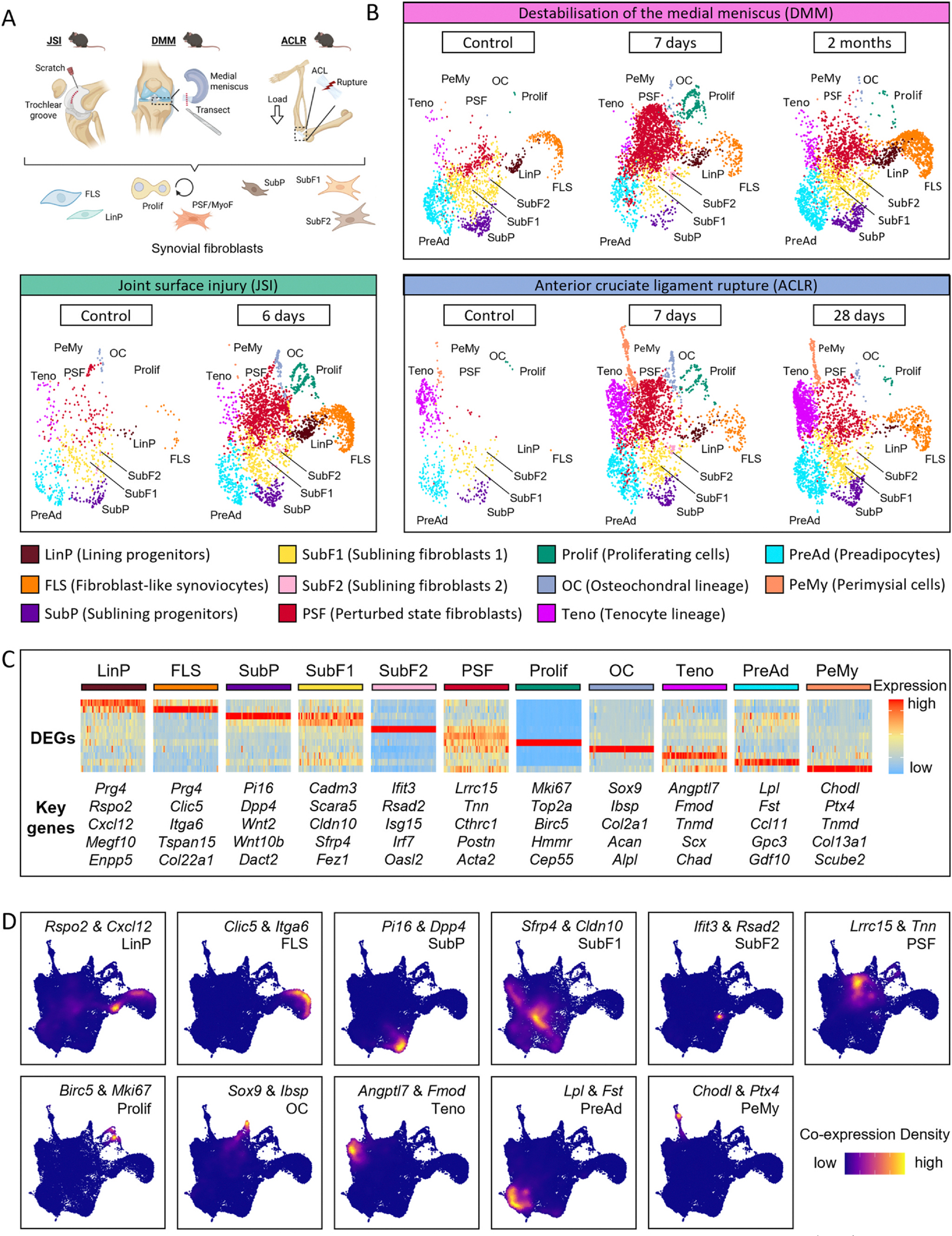
Harmonisation of synovial fibroblast populations across mouse models of joint injury. ScRNA-seq data of synovial fibroblasts from three different mouse models of joint injury were integrated for analysis. (A) Schematic representation of the joint injury models and synovial fibroblast populations analysed in the current study. (B) Unsupervised UMAP plots downsampled to show equal cell numbers (2875 cells) for all samples from injured mice to facilitate visual comparisons. (C) Differentially expressed genes (DEGs) used to identify each cluster. Heatmaps show expression of top 50 DEGs per cluster. Key genes specify selected DEGs that identify each cell type. (D) Nebulosa plots showing combined expression, based on kernel density estimation, of two cluster-defining genes. See Supplementary Fig. 1B for Nebulosa plots showing individual genes. LinP, lining progenitor cells; FLS, fibroblast-like synoviocytes; SubP, sublining progenitor cells; SubF, sublining fibroblasts; PSF, perturbed-state fibroblasts; Prolif, proliferating cells; OC, osteochondral lineage cells; Teno, tenocyte lineage cells; PreAd, pre-adipocytes; PeMy, perimysial cells.

To comparatively investigate synovial fibroblast responses to different types of injury, we integrated scRNA-seq data from independent studies employing a surgical joint surface injury (JSI) model that elicits a repair response analysed at day 6 [12], a surgical model of joint instability due to resection of the medial meniscotibial ligament causing destabilisation of the medial meniscus (DMM) analysed at day 7 and two months [13], and a non-surgical model of joint instability due to excessive load causing anterior cruciate ligament rupture (ACLR) analysed at day 7 and day 28 [14] (Fig. 1A; Supplementary Tables 1 and 2). Both DMM and ACLR lead to a progressive OA-like phenotype characterised by cartilage damage, osteophyte formation, and synovitis. ACLR leads to more rapid OA progression compared to DMM, and the late time points analysed in this study represent established OA in both models [25,26].

Unsupervised computational integration of datasets using reciprocal PCA, after excluding poor quality cells, haematopoietic and endothelial cells, yielded 30,339 total cells across 11 clusters, with number of cells per experimental group ranging from 1012 to 8165 (Fig. 1B,C; Supplementary Table 2 and 3). Clustering level was set based on analysis of cluster-specific gene expression patterns (Fig. 1B,C; Supplementary Table 4) to identify cell types with previously ascribed functions without overfitting. Synovial lining fibroblasts, characterised by high expression of *Prg4* (Supplementary Fig. 1A), separated in all three models into an immature LinP population [12] and FLS (Fig. 1B,C). Synovial sublining fibroblasts, characterised by expression of *Thy1* and *Cd34* (Supplementary Fig. 1A), included *Dpp4*-expressing SubP cells [12–14,20] and two other fibroblast populations (SubF1 and SubF2) (Fig. 1B,C). In addition, a population expressing markers of perturbed-state fibroblasts (PSF), including *Lrrc15* and *Tnn* [4,12], and the myofibroblast markers *Acta2* and *Tagln* [4,8], as well as a population of proliferating cells (Prolif), expanded after injury in all models (Fig. 1B,C). Finally, osteochondral-lineage cells (OC), tenocyte-lineage cells (Teno), pre-adipocytes (PreAd), and a population of cells primarily present in the ACLR dataset that resemble perimysial (PeMy) cells [27] were identified (Fig. 1B,C). While some cell populations could be readily defined based on a single marker, others (most notably the PSF cluster) showed a less distinct marker profile (Fig. 1C; Supplementary Fig. 1B). Here, two markers were identified for each cell population that, in combination, provided good discriminatory ability (Fig. 1D; Supplementary Fig. 1B).

### Lining fibroblast expansion and perturbed-state phenotypes after injury

**Fig. 2:**
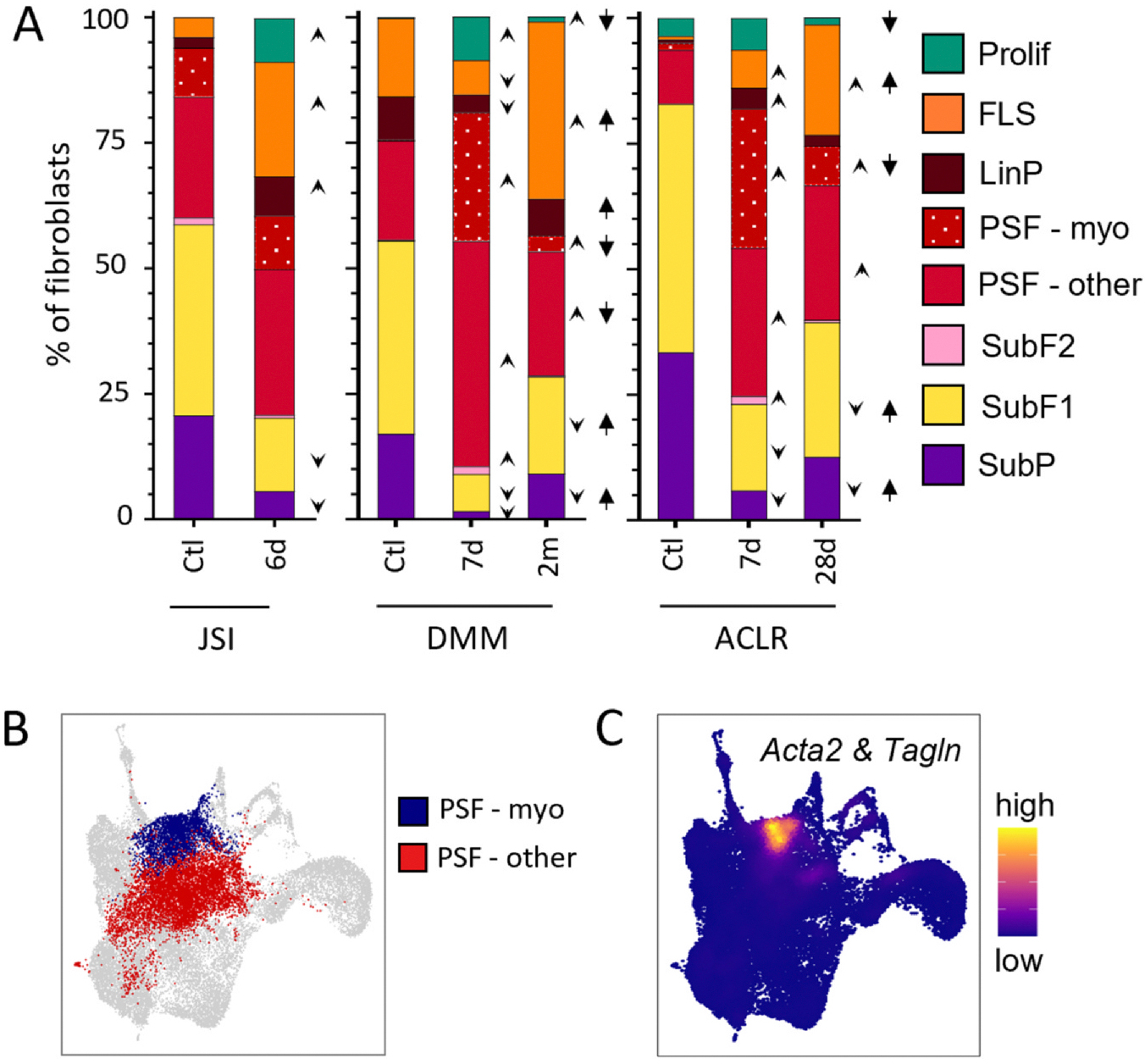
Dynamics of synovial fibroblast expansion and myofibroblast differentiation in response to injury. (A) Relative abundance of synovial fibroblast populations in the three different injury models. (⮝) indicates *p* < 0.0001 increase vs control, (⮟) indicates *p* < 0.0001 decrease vs control, (🠝) indicates *p* < 0.0001 increase vs 7d and (🠟) indicates *p* < 0.0001 decrease vs 7d based on Pearson’s Chi-squared test followed by pairwise Pearson’s Chi-squared test with FDR correction. (B) UMAP plot showing PSF subsets identified by sub-clustering of the PSF population, mapped onto the integrated data from all injury models. (C) Nebulosa plot showing the combined expression, based on kernel density estimation, of the myofibroblast markers *Acta2* and *Tagln*. LinP, lining progenitor cells; FLS, fibroblast-like synoviocytes; SubP, sublining progenitor cells; SubF, sublining fibroblasts; PSF, perturbed-state fibroblasts; Myo, myofibroblasts; Prolif, proliferating cells.

To compare the cellular response to injury between the three models, we first analysed relative abundances of fibroblast populations (Fig. 2A). To account for differences in cell isolation protocols between studies, we compared each injured state to its respective steady-state control. The proportion of PSF greatly increased at early stage in the DMM (3.5-fold, *p* < 0.0001) and ACLR models (4.7-fold, *p* < 0.0001), while their relative expansion was modest and not statistically significant after JSI (1.2-fold) (Fig. 2A; Supplementary Table 4). Sub-clustering of the PSF population revealed a subset of myofibroblasts, characterised by co-expression of *Acta2* and *Tagln* (Fig. 2B,C; Supplementary Table 5). Similarly, the proportion of myofibroblasts greatly increased after DMM (108-fold, *p* < 0.0001) and ACLR (21-fold, *p* < 0.0001), while it barely changed after JSI (1.1-fold) (Fig. 2A). The myofibroblast expansion largely resolved at late stage following DMM and ACLR (Fig. 2A), indicating this to be a dynamic, transient response to injury despite OA progression in both models. The response to JSI was characterised instead by rapid increases in the proportions of LinP cells (3.8-fold, *p* < 0.0001) and FLS (5.7-fold, *p* < 0.0001), which was not observed at early stage after DMM (Fig. 2A), consistent with the findings from the original studies [12–14]. However, FLS abundance increased in both DMM and ACLR models at late stage (Fig. 2A). Taken together, these data indicate that the early response to acute cartilage injury in the JSI model is comparatively dominated by expansion of lining fibroblasts (LinP cells and FLS), while mechanical instability leading to progressive cartilage damage and synovial fibrosis in the DMM and ACLR models of OA induced transient myofibroblast differentiation followed over time by FLS expansion.

### Shifts in transcriptomic profiles following injury

**Fig. 3:**
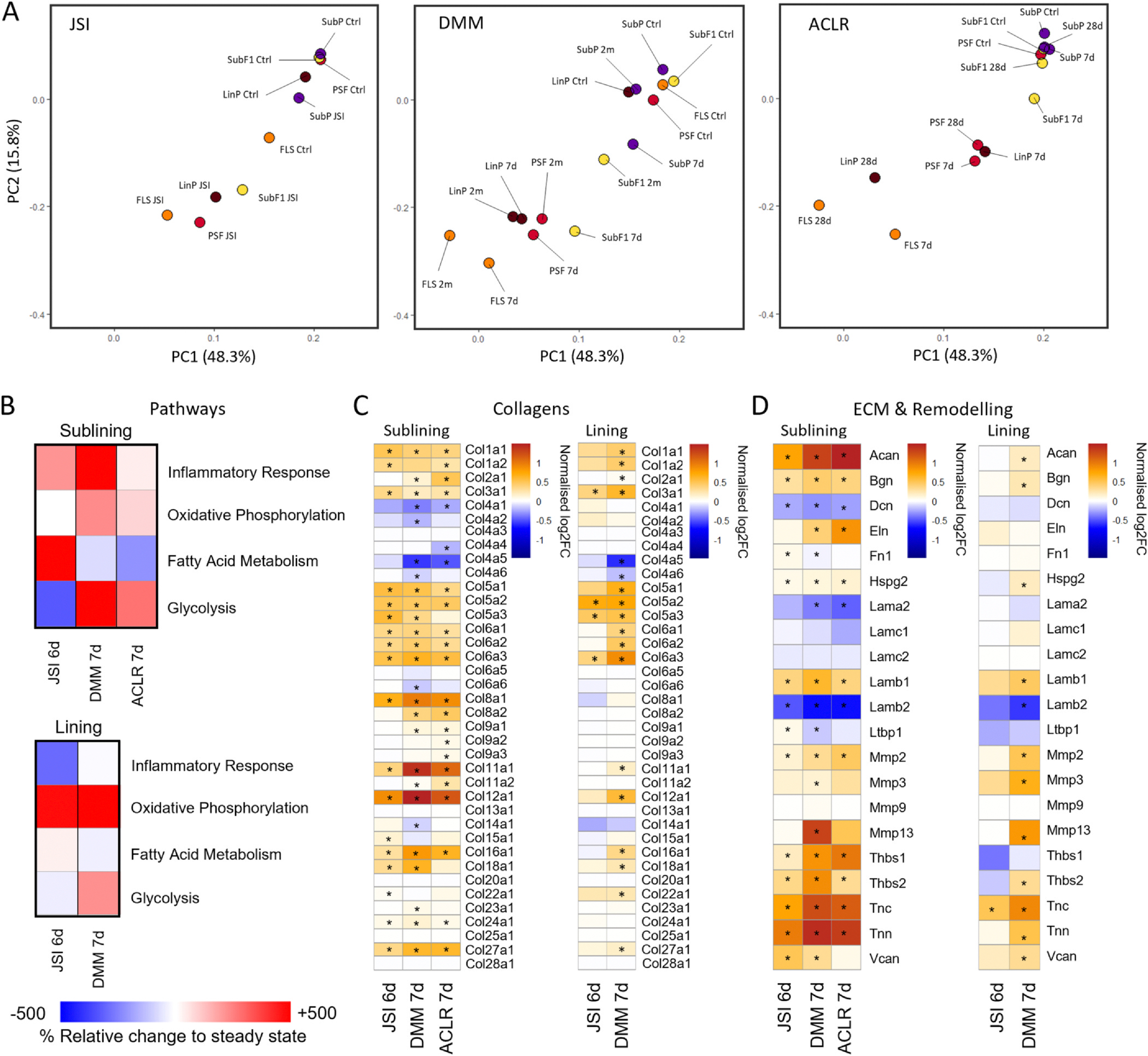
Transcriptomic shifts in synovial fibroblasts in response to injury. (A) PCA plots of integrated synovial fibroblast cluster transcriptome similarity based on Reactome pathway analysis, in steady-state (control) and injured conditions, separated by model for visualisation. FLS and LinP populations from control samples in the ACLR dataset, and SubF2 populations from all samples in all datasets, were excluded from the analysis due to very low or absent cell numbers. (B) GSVA analysis of Hallmark pathways in sublining (SubP, SubF1, SubF2 and PSF) and lining fibroblasts (LinP and FLS). Enrichment was normalized to respective controls to show relative change in response to injury. Lining fibroblasts from the ACLR dataset were excluded due to very low cell numbers in the control sample. (C-D) Heatmaps showing changes in expression of (C) collagens and (D) extracellular matrix (ECM) and remodeling genes in sublining and lining fibroblasts. Log2-fold-change in gene expression was normalized to respective controls to show relative change in response to injury. * indicates *p* < 0.05 compared to control, non-parametric Wilcoxon rank sum test with Bonferroni correction. LinP, lining progenitor cells; FLS, fibroblast-like synoviocytes; SubP, sublining progenitor cells; SubF, sublining fibroblasts; PSF, perturbed-state fibroblasts.

Next, we performed Reactome pathway analysis on the integrated dataset and used PCA to project the aggregated pathway analyses onto 2D space to provide an unbiased, high-level visual comparison of transcriptional programs for each cell population and the degree to which they were altered by injury (Fig. 3A). The pathway analyses revealed transcriptomic shifts in all populations after injury, with the SubP population showing least divergence from steady-state control in all datasets. In addition, pathway-level transcriptomic changes in SubP and SubF1 populations appeared transient, whereas they continued to persist at late stage in the PSF population as well as the lining fibroblasts (both LinP cells and FLS), indicative of a sustained perturbed phenotype during OA (Fig. 3A).

Inflammation and metabolic dysfunction play a key role in OA pathogenesis [28,29]. We therefore investigated these pathways in synovial fibroblasts by performing gene-set variation analysis (GSVA) [30]. The fibroblast subpopulations were combined into a lining (LinP cells and FLS) and sublining population (SubP, SubF1, SubF2, PSF) to ensure sufficient cell numbers in control samples for meaningful data. Sublining fibroblasts showed enrichment of the Inflammatory Response pathway after injury, especially in the JSI and DMM models, possibly related to the surgery (Fig. 3B). This was not observed in the lining fibroblasts (Fig. 3B). Fibroblasts in all models and across lining and sublining populations showed enrichment of the Oxidative Phosphorylation pathway in response to injury (Fig. 3B), reflecting high mitochondrial activity and energy production associated with fibroblast activation [31]. However, the Fatty Acid Metabolism pathway was enriched in the JSI model, while the Glycolysis pathway was enriched in DMM and ACLR (Fig. 3B), suggesting a difference in energy source utilised.

Next, we sought to investigate changes in genes associated with fibroblast activity, focusing on collagen genes (Fig. 3C) and other genes involved in extracellular matrix production and remodeling (Fig. 3D), known to be upregulated in OA [32]. Relative to their steady-state controls, lining and sublining fibroblasts, particularly in DMM and ACLR models, showed marked upregulation of collagens and extracellular matrix components associated with myofibroblast activity [3,33], as well as increased expression of genes encoding matrix metalloproteinases (MMPs), consistent with a more catabolic environment [34] (Fig. 3C,D; Supplementary Table 6).

### Molecular regulation of the perturbed-state fibroblasts

**Fig. 4:**
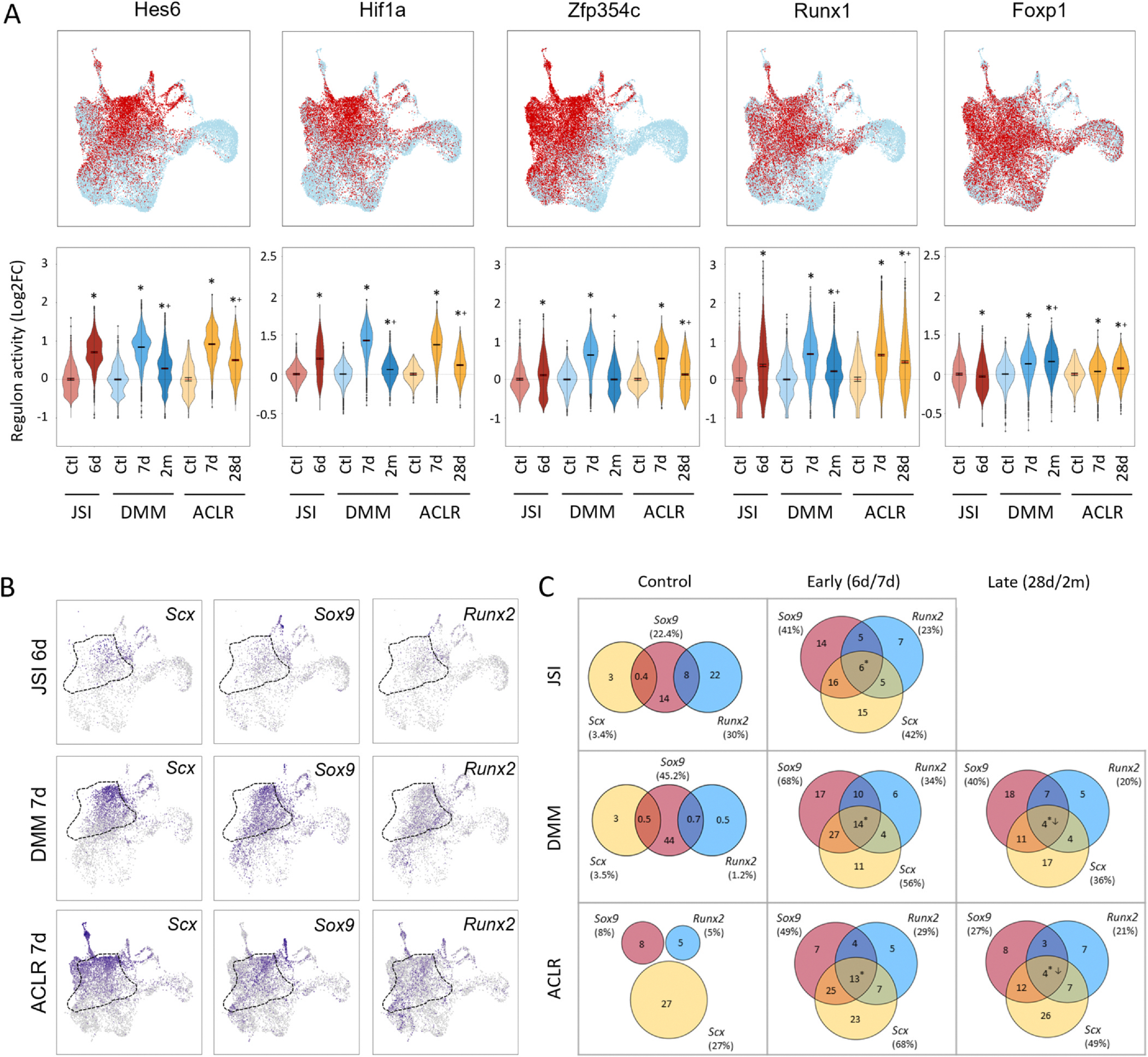
Molecular regulation of the perturbed-state fibroblast phenotype. (A) UMAP plots of integrated steady-state (control) and injured datasets showing binary activity of regulons active in more than 50% of cells in the PSF cluster, with lower activity in other cell clusters. Violin plots of regulon activity within synovial fibroblasts (excluding the proliferating cell cluster), normalised to the respective steady-state control (Ctl), are shown underneath. Regulon activity was calculated from the AUCell score (scale 0–1) and presented as log2-fold-change. Line and error bars indicate mean and 95%CI. * indicates *p* < 0.001 vs control and + indicates *p* < 0.001 vs 7d based on MANOVA with pairwise comparison and Benjamini-Hochberg correction. (B) UMAP plots showing expression of tenocyte (*Scx*), chondrocyte (*Sox9*) and osteoblast (*Runx2*) lineage-specifying transcription factors. Dotted outline indicates the approximate border of the PSF population. (C) Venn diagrams showing percentage of cells in the PSF cluster expressing *Scx*, *Sox9* and *Runx2*. Analysis of triple positive cells was performed by Pearson’s Chi-squared test followed by pairwise Pearson’s Chi-squared test with FDR correction. * indicates *p* < 0.0001 vs control and ↓ indicates *p* < 0.0001 vs 7d. LinP, lining progenitor cells; FLS, fibroblast-like synoviocytes; SubP, sublining progenitor cells; SubF, sublining fibroblasts; PSF, perturbed-state fibroblasts.

To analyse regulon activity underpinning the PSF response, we used the SCENIC package to identify regulons that were highly active within the PSF cluster and showed lower activity in other clusters. Analysis on the integrated dataset revealed Hes6, Hif1a, Zfp354c, Runx1 and Foxp1 regulons to be associated with the perturbed-state phenotype (Fig. 4A; Supplementary Table 7). The activities of Hes6, Hif1a, Zfp354c and Runx1 regulons were upregulated at early stage after injury compared to control in all models, and partly returned to steady-state levels at late stage in both DMM and ACLR models. The JSI model showed greater variation, with Hes6 robustly upregulated, while Zfp354c upregulation was modest. In contrast, Foxp1 regulon activity progressively increased after both DMM and ACLR (Fig. 4A; Supplementary Table 7). This indicates that the regulons that underpin the perturbed-state phenotype that emerges at early stage after injury and those that drive the persistently perturbed phenotype in established OA are at least partly distinct.

We previously reported that PSF in JSI and serum-transfer-induced inflammatory arthritis models, while differing in their perturbed phenotypes, share the adoption of multilineage potency [12]. To determine whether this is also true for other models of joint injury, we analysed the PSF population for co-expression of the transcription factors *Sox9* (chondrocyte-lineage), *Runx2* (osteoblast-lineage) and *Scx* (tenocyte-lineage) (Fig. 4B,C). While we did not detect co-expression of all three transcription factors within individual PSF from control mice, co-expression of these lineage-specifying transcription factors emerged after JSI (6% of PSF, *p* < 0.0001), DMM (14% of PSF, *p* < 0.0001) and ACLR (13% of PSF, *p* < 0.0001) (Fig. 4B,C). This persisted, although at reduced frequency, at late stage after DMM (4% of PSF, *p* < 0.0001 vs control; *p* < 0.0001 vs early stage) and ACLR (4% of PSF, *p* < 0.0001 vs control; *p* < 0.0001 vs early stage) (Fig. 4C), indicating that some PSF remain in an activated state with multilineage potential.

### Proliferation dynamics and trajectories underpinning synovial fibroblast expansion

**Fig. 5:**
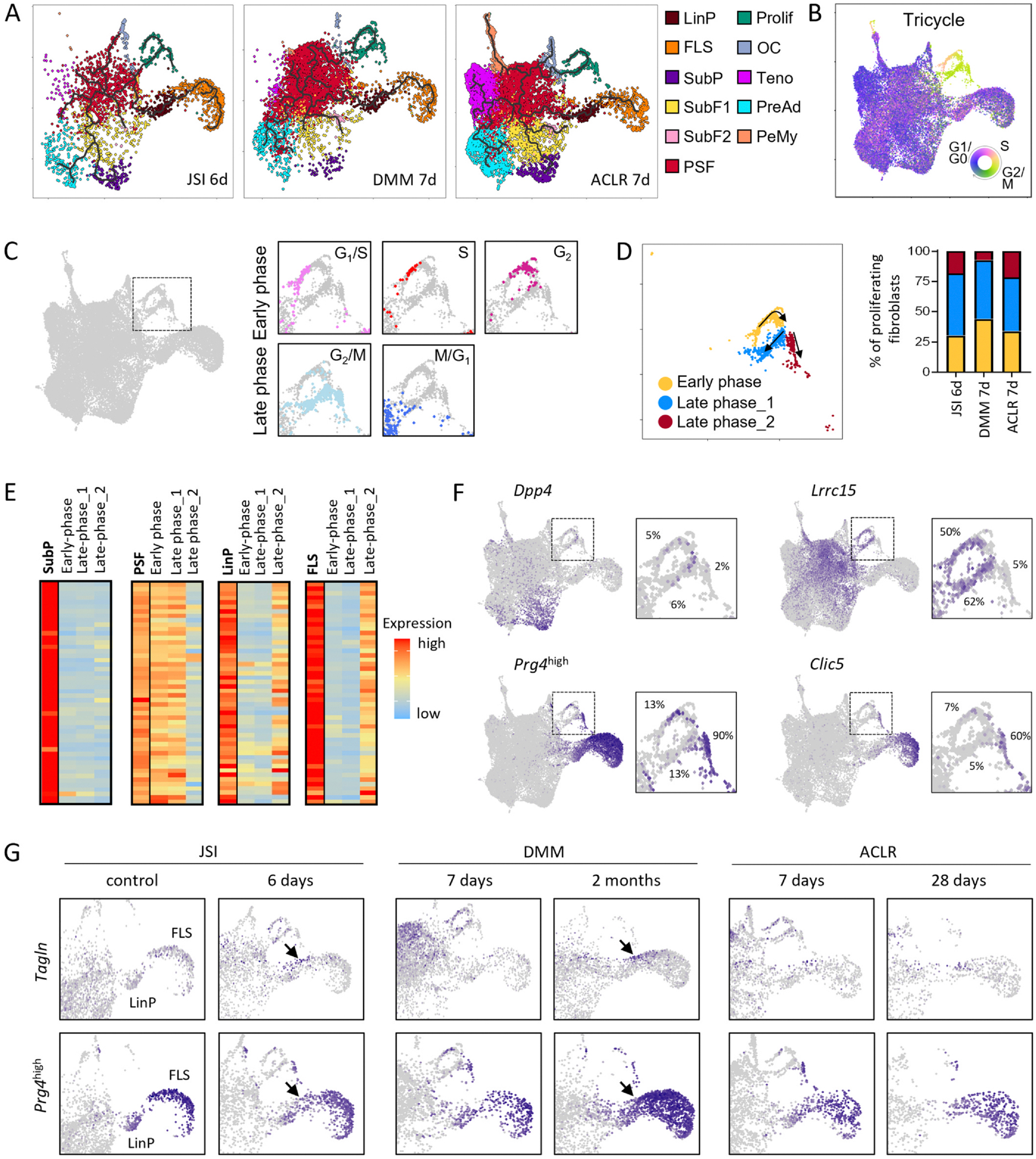
Perturbed-state fibroblast and FLS proliferation after injury. (A) Monocle 3 pseudotime trajectory analysis applied to samples from injured mice showing inferred lineage trajectories. (B-F) Cell cycle analysis performed on integrated data from JSI 6d, DMM 7d, and ACLR 7d samples. (B) UMAP plot showing cell cycle position based on scoring by the Tricycle package. (C) Visualization of predicted cell cycle phases within the proliferating cells. Dotted outline on full UMAP indicates enlarged region on the right. (D) UMAP plot of the proliferating cluster after supervised sub-clustering according to cell cycle phase as shown in (C). Stacked bar chart shows percentage of cells within each cell cycle phase. (E) Heatmaps showing expression of top 50 DEGs of the SubP, PSF, LinP and FLS clusters by each of the proliferating sub-clusters. See Supplementary Fig. 2 for heatmaps of all clusters. (F) UMAP plots showing expression of indicated genes. *Prg4*^high^ plot shows cells with an expression level greater than 4. Dotted outline indicates enlarged region on the right to highlight expression in the proliferating sub-clusters. Percentages indicate proportion of cells within each sub-cluster (early phase, late phase 1 or late phase 2) expressing indicated gene. (G) UMAP plots showing expression of *Tagln* (expression level greater than 1) and *Prg4*^high^ (expression level greater than 4) by cells in the lining fibroblast (LinP and FLS) clusters. Arrows indicate cells co-expressing *Tagln* and *Prg4*. LinP, lining progenitor cells; FLS, fibroblast-like synoviocytes; SubP, sublining progenitor cells; SubF, sublining fibroblasts; PSF, perturbed-state fibroblasts; Prolif, proliferating cells; OC, osteochondral lineage cells; Teno, tenocyte lineage cells; PreAd, pre-adipocytes; PeMy, perimysial cells.

To understand how proliferation underpins PSF and FLS expansion, we first performed trajectory analysis. In all models at early stage post-injury, inferred trajectories indicated PSF as a ‘central node’, suggesting their plasticity to transition between different cell states (Fig. 5A). In the ACLR and DMM models, inferred trajectories connected proliferating cells to the myofibroblast subset, while in the JSI model, inferred trajectories connected proliferating cells to PSF and lining fibroblast populations (Fig. 5A). We next employed cell cycle analysis to understand proliferation dynamics. Due to the low number of proliferating cells, we focused on the integrated data at early stage post-injury. This in-depth analysis revealed progression of cells through the cell cycle along early and late cell-cycle phase trajectories (Fig. 5B,C). Manual sub-clustering of proliferating cells (Fig. 5D) and pseudo-bulk analysis (Fig. 5E; Supplementary Fig. 2, Supplementary Table 8) showed divergence between late-phase cells, with one population transcriptomically resembling PSF and another resembling lining fibroblasts (LinP/FLS). Visualisation of marker gene expression confirmed the separation between proliferation trajectories, showing that cells displayed an activated PSF or lining fibroblast phenotype as they emerged from the cell cycle (Fig. 5F). These findings are consistent with our previous report in the JSI model that identified two populations of proliferating cells in lining and sublining synovium, respectively [12], and indicates similar dynamics after DMM and ACLR, although the proportion of cells in the lining fibroblast proliferation trajectory was low in the DMM model (Fig. 5D). While the transcriptome alone is not sufficient to determine the origin of the proliferating cells, transcriptomic similarity suggests that the pool of PSF fed into the proliferation trajectory, with a contribution from *Prg4*^high^ LinP cells (Fig. 5E,F). Conversely, their transcriptomic divergence from *Dpp4+* SubP cells (Fig. 5E,F) suggests that the SubP cells did not directly feed into the proliferating population at the timepoints analysed. In addition to the two proliferation trajectories supplying new PSF and lining fibroblasts, respectively, we observed the appearance of cells with an intermediate phenotype that expressed both the myofibroblast marker *Tagln* and, at high levels, the lining fibroblast marker *Prg4*. These cells were present in the JSI model at early stage, while they appeared at late stage in the DMM model (Fig. 5G). Together with the dynamics of relative cellular abundance (Fig. 2A), these data support a model whereby fibroblasts in synovium can adopt an activated phenotype and proliferate after injury to expand both the PSF and lining fibroblast populations, with a further feeding of PSF into the FLS differentiation trajectory, with relative dynamics depending on injury model and stage.

## Discussion

Synovial fibroblasts play pivotal roles following injury and in arthritis. They proliferate and produce factors that mediate inflammation and tissue damage [35], while they also include cells with stem and progenitor capabilities that promote repair [15]. Single-cell studies are fundamental to deciphering these distinct roles of synovial fibroblasts across various joint conditions, aiding in the development of targeted therapies. In rheumatoid arthritis research, seminal scRNA-seq studies employing mouse models of immune-mediated inflammatory arthritis and synovium from RA patients have identified pathogenic synovial fibroblast subsets responsible for driving inflammation and causing bone and cartilage damage [6,8,36,37]. In OA research, disrupted biomechanics is a significant risk factor in disease pathogenesis. Hence, various mouse models of acute injury and post-traumatic OA have been used to study the transcriptional and functional changes in synovial fibroblasts and elucidate their roles in joint repair and OA progression. Here, we sought to compare cellular and molecular responses of synovial fibroblasts in relation to type and duration of injury. We find commonalities and crucial differences in the response to different types of injury at early stage. Acute focal injury to the cartilage (JSI model), which is associated with a cartilage repair response [16], triggered rapid lining fibroblast expansion. In contrast, ligament injury causing joint destabilisation and OA-like damage (DMM and ACLR models) was dominated by fibroblast activation into a perturbed state, with a subset adopting a myofibroblast phenotype, followed by lining fibroblast expansion over time. This is consistent with a recent study in humans observing progressive lining hyperplasia and a pro-fibrotic lining fibroblast transcriptome in OA [32]. Of note, the JSI and DMM models are both surgical models with a wound healing component resulting from arthrotomy, while the ACLR model is non-surgical and involves rupture of the ACL due to excessive mechanical load. Notwithstanding an influence from surgical wound healing, the similarity in response between DMM and ACLR indicates that the dominant factor driving the early synovial fibroblast response in these models may be altered mechanical load due to joint instability. Conversely, a main driver of the expansion of the lining fibroblasts appears to be damage to the articular cartilage, which in the JSI model is acute while in the DMM and ACLR models it occurs gradually and progressively.

Trajectory analyses connected the PSF to all other stromal cell populations in the joint. Lineage-tracing experiments point to a dual origin of PSF from lining and sublining populations, with relative contributions likely determined by type and severity of insult. In the DMM model, progeny from both *Dpp4*-expressing sublining cells and *Prg4*-expressing lining cells were found to expand throughout synovium and to give rise to *Scx*-expressing and α-Smooth-muscle-actin-expressing PSF [13]. At the same time, the PSF are also poised to undergo differentiation to osteoblasts, chondrocytes and tenocytes, as indicated by co-expression of the lineage-specifying transcription factors *Runx2*, *Sox9* and *Scx,* respectively. Accordingly, lineage-tracing experiments have shown that *Prg4*-expressing lining progenitors give rise to chondrocytes that form chondro-osteophytes after DMM [19] and repair cartilage after osteochondral injury [18]. We show here that the injury-induced co-expression of lineage-specifying transcription factors is a common phenomenon independent from type of injury. Since this phenomenon was also observed in immune-mediated inflammatory arthritis [12], we infer that the adoption of an activated phenotype capable of multilineage specification and possibly differentiation is a universal response of synovial fibroblasts to insult.

Activity of transcriptional programmes underpinning the PSF phenotype varied depending on the model and stage of injury response. Elucidating their functions in synovial fibroblasts may help to shed light on the mechanisms of repair *versus* progression towards OA following injury. Hif1α is a transcription factor induced under hypoxic or inflammatory conditions and involved in regulating fibroblast extracellular matrix remodeling and wound healing [38], which could underpin the increased expression of collagen and other matrix-related genes in response to injury. *Zfp354c* (*ZNF354C* in humans), previously identified from synovial microarray data as associated with OA [39], is a transcriptional repressor with unclear functions. Its transient upregulation predominantly in the post-traumatic OA models indicates it may play a role in the myofibroblast response. Foxp1 regulon activity, which progressively increased in the post-traumatic OA models, has been shown to enhance fibrosis [40] and regulate endochondral ossification [41], suggesting it has an important role in OA progression and osteophyte formation [42].

Synovial hyperplasia in response to injury is driven by proliferation, which was transient in all injury models. We observed that differentiation towards either a PSF or a lining fibroblast phenotype occurs as cells complete the cell cycle. These findings are consistent with the existence of both sublining and lining proliferating populations after acute cartilage injury [12,42], and the data presented here indicates this also applies to the DMM and ACLR models, with relative abundance seemingly dependent on model. As we reported previously, in steady state, a slow rate of lining fibroblast turnover to maintain homeostasis is sustained by a pool of lining progenitors, while the rapid expansion of lining fibroblasts following acute cartilage injury involves contribution from activated sublining fibroblasts [12]. Whether activated sublining fibroblasts i) enter the cell cycle and directly differentiate into FLS, ii) transition to a lining progenitor phenotype prior to cell cycle entry, and/or iii) differentiate into lining fibroblasts independently from proliferation remains to be determined. We observed the appearance of cells with an intermediate phenotype between PSF and FLS in the JSI model at early stage and the DMM model at late stage. This observation is consistent with a direct transition of PSF to lining fibroblasts, and could explain the dramatic shift in relative abundance of PSF and FLS between early and late timepoints post-DMM, despite the proliferative response being transient.

Lineage tracing of *Dpp4*-expressing SubP cells showed that their offspring proliferate, expand, and give rise to activated PSF after DMM [13]. Our analyses indicate that their direct contribution to proliferating cells one week after injury is minimal in any of the injury models, with proliferation and expansion at this point driven by the pool of PSF with a contribution from LinP cells. It is, however, possible that proliferation of the SubP cells preceded the timepoints analysed. Meanwhile, the remaining SubP population showed relatively modest transcriptomic divergence after injury, indicating that a pool of SubP cells may be preserved and protected from the effects of injury at the transcriptome level. This could be due to protecting niche factors that shield them from injury signals, which could point to a process of self-renewal. Taken together, these data support a model whereby multiple fibroblast populations adopt an activated phenotype and proliferate to expand both the sublining and lining fibroblast populations, with a further feeding of activated sublining fibroblasts into the FLS differentiation trajectory, depending on injury model and stage.

In summary, single-cell analysis of synovium at multiple time points in mouse models of injury has allowed in-depth characterisation of myofibroblasts, commonly linked with fibrotic outcomes and progression to end-stage OA, and offered an opportunity to identify and study fibroblasts with stem/progenitor cell activity at the early stage of response to injury, opening the exciting prospect of targeted regenerative therapies in OA. The commonalities of a repair response across injury models at the early stage support the emerging concept that an early intervention that targets intrinsic repair combined with ablation of the deleterious fibroblast populations as they emerge, could be a valid treatment strategy in OA [6,36,44].

## Supplementary Material

1

2

3

4

5

6

## Data Availability

ScRNA-seq data analysed in this study are available in a public, open access repository. All data supporting the findings of this study are available within the Article and its Supplementary Information files.

## Appendix A. Supporting information

Supplementary data associated with this article can be found in the online version at doi:10.1016/j.joca.2025.12.023.
