# Supplementary material for: Synovial fibroblast responses to different types of injury resulting in cartilage repair or osteoarthritis
Source: Osteoarthritis Cartilage. Author manuscript; Available in PMC 2026 Jul 7. (PMC13338840; doi:10.1016/j.joca.2025.12.023)
Supplement: 1 [file NIHMS2178807-supplement-1.docx]

Supplementary Materials for

**Synovial fibroblast response to different types of injury resulting in cartilage repair or osteoarthritis**

**Authors:**

Fraser L. Collins ^#1^, Alexander J Knights ^#2,3,4^, Tristan Maerz ^2,5,6^, Anke J. Roelofs *^1^, Cosimo De Bari *^1^

**Author Affiliations:**

^1^Rheumatology Research Group, Institute of Genetics and Cancer, University of Edinburgh, UK;

^2^Department of Orthopaedic Surgery, University of Michigan, Ann Arbor, MI, USA;

^3^Department of Orthopaedic Surgery, Washington University, St Louis, MO, USA;

^4^Center of Regenerative Medicine, Washington University, St Louis, MO, USA;

^5^Institute for Biomechanics, Department of Health Sciences and Technology, ETH Zürich, Zürich, Switzerland

^6^Departments of Biomedical Engineering and Internal Medicine (Division of Rheumatology), University of Michigan, Ann Arbor, MI, USA;

# FLC and AJK contributed equally to this work; * AJR and CDB contributed equally to this work.

**Correspondence:**

Cosimo De Bari, Institute of Genetics and Cancer, University of Edinburgh, Crewe Road South, Edinburgh EH4 2XU, United Kingdom. Email: [cosimo.debari@ed.ac.uk](mailto:fraser.collins@ed.ac.uk).

**This PDF file includes:**

Extended Methods

Supplementary Figures 1 to 2

Supplementary Tables 1 to 3

**Extended Method**

**Mouse models and cell isolation procedures**

For each dataset analysed in this study, the mouse models and cell isolation procedures are detailed in the original publications (1–3) and summarised in Supplementary Table 1. Briefly, the JSI dataset was obtained by collagenase digestion of knees from *Gdf5-Cre*;*tdTom*;*Pdgfrα-H2BGFP* mice 6 days after JSI surgery, in which a scratch is made in the cartilage of the femoral groove, with control mice not undergoing surgery. This was followed by fluorescence-activated cell sorting of *Gdf5*-lineage fibroblasts (co-expressing tdTomato and GFP) and non-*Gdf5*-lineage fibroblasts (expressing only GFP) (1). Data from the two sorted fibroblast populations were integrated for the current study. The DMM dataset was obtained from wild-type mice at either 7 days or 2 months after DMM surgery, in which the medial menisco-tibial ligament is resected, with control mice not undergoing surgery. Synovium was dissected and digested with an initial trypsin digestion followed by a collagenase digestion. Cd45 fluorescence-activated cell sorting was used to enrich the fibroblast population, with a proportion of the Cd45+ cells added back in to the sample (2). The ACLR dataset was obtained from wild-type mice at either 7 days or 28 days after excessive loading of the knee causing ACL rupture, with control mice undergoing anaesthesia without loading, by dissecting the synovium followed by collagenase and liberase digestion (3).

**Reactome analysis**

Reactome analysis was performed using the R package ReactomeGSA (4). Briefly, pathway-level expression values were generated for identified clusters by calculating their mean gene expression, followed by gene-set variation analysis using the ‘analyse_sc_clusters’ function. Principal component analysis (PCA) of the pathway-level expression was performed using the ‘plot_gsva_pca’ function.

**GSVA Hallmark pathway analysis**

The R package GSVA (5) was used to analyse Hallmark pathway enrichment. Gene sets were downloaded from the Molecular Signature Database (MSigDb) (6,7). Cells were clustered into sublining or lining fibroblasts and a pseudo-bulk dataset was generated using the Seurat ‘AggregateExpression’ function. A parameter object was created using the ‘gsvaParam’ function and pathway activity was calculated with the ‘gsva’ function. Samples were normalised by quantile normalisation and relative changes in pathway enrichment to respective steady-state control were calculated and plotted using the R package pheatmap.

**Gene regulatory network analysis**

Regulon analysis was performed using the SCENIC package for R (8). The expression matrix was isolated from the Seurat object and the co-expression gene regulatory network built using GRNBoost. Transcription factor motifs were created using RcisTarget and the mm9-tss-centred-10kb (mouse) database. AUCell was used to calculate regulon activity scores, which were normalized to the respective steady-state control (1).

**Trajectory and cell cycle analysis**

Trajectories were computed using the Monocle3 (9) R package. Pre-computed cell embeddings and clusters from the Seurat pipeline served as input. Gene expression data were isolated from the Seurat object using the Seurat ‘GetAssayData’ function and a Monocle cell dataset (cds) created using the Monocle3 ‘new_cell_data_set’ function. The cds was pre-processed, dimensions were reduced, and cells were clustered using the Monocle3 ‘preprocess_cds’, ‘reduce_dimension’ and ‘cluster_cells’ functions. Trajectories were determined using the Monocle ‘learn_graph’ function. Orthogonal inference and visualization of the cell cycle process was performed using the R package Tricycle (10), and the inbuilt cell cycle stage predictor based on the method by Schwabe *et al*. (11).


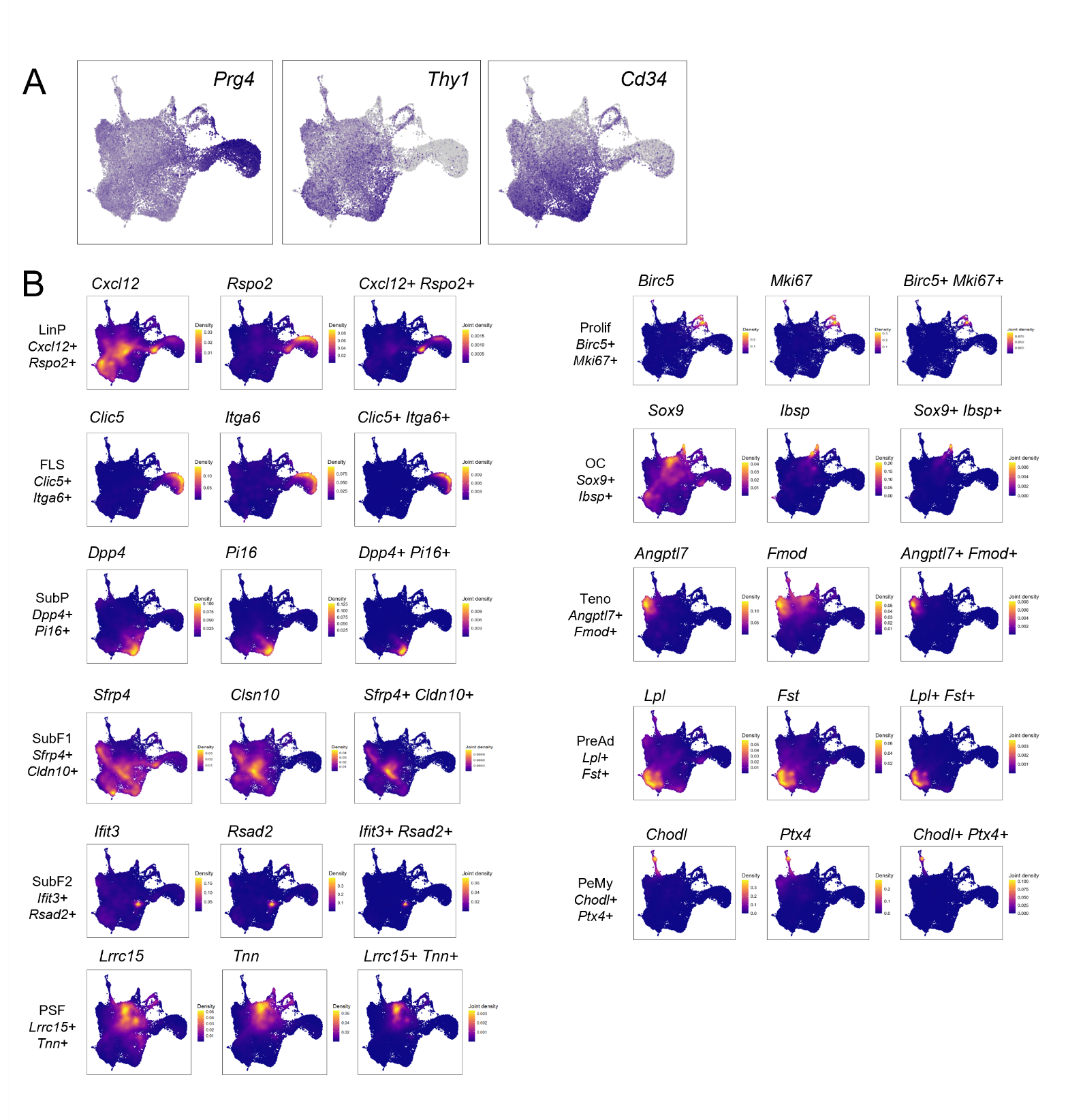


**Supplementary Figure 1. Identification of cell sub-populations**. (A) UMAP plots of integrated data from all injury models showing expression of the synovial lining fibroblast marker *Prg4*, and the synovial sublining fibroblast markers *Thy1* and *Cd34*. (B) Side-by-side Nebulosa plots of integrated data showing individual and combined marker gene expression for each cell population (extended data for Fig. 1D).


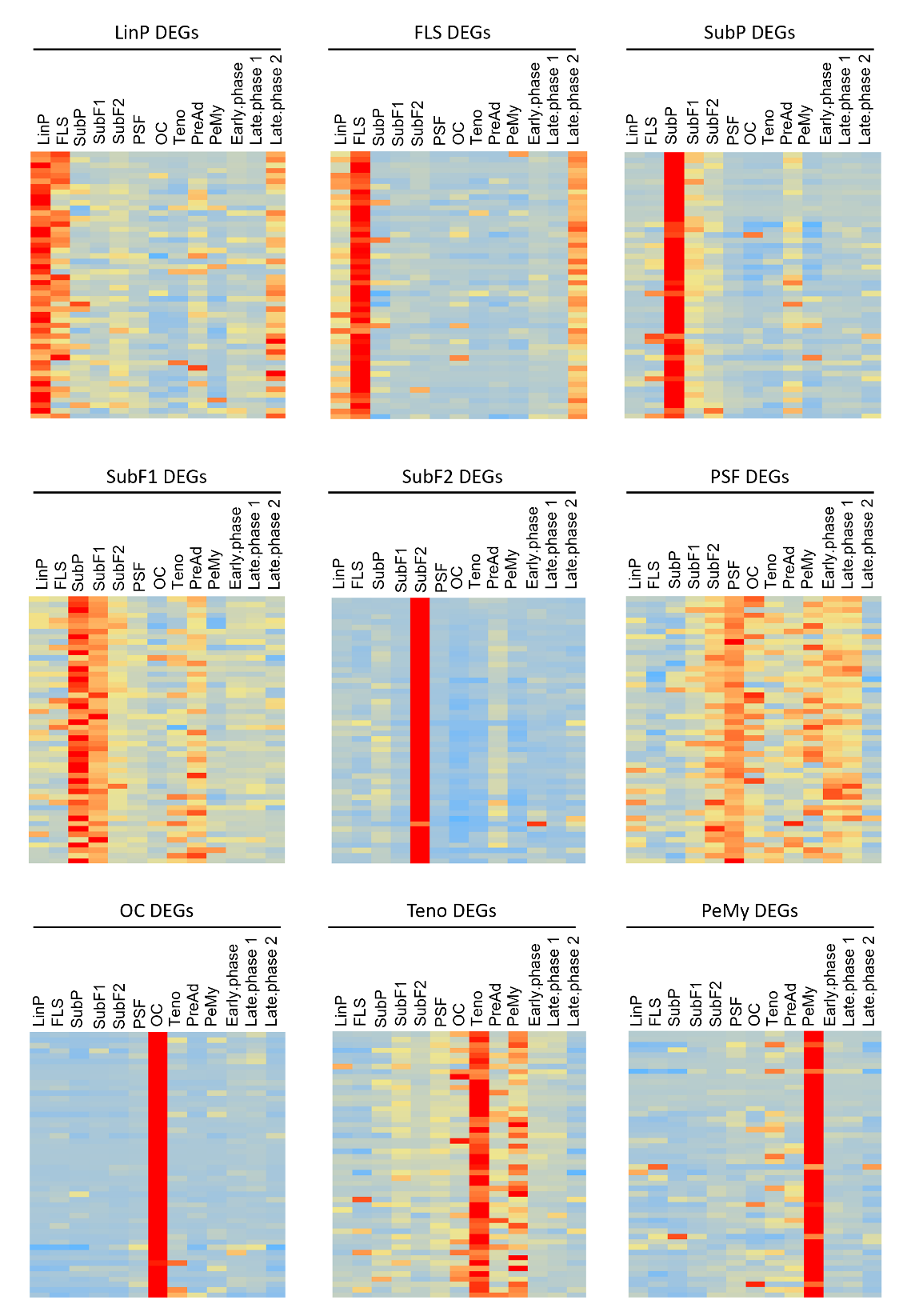


**Supplementary Figure 2. Proliferating cell sub-cluster transcriptomes.** Heatmaps showing average gene expression for the top 50 DEGs of each of the identified clusters to identify similarities with the proliferating cell sub-clusters. LinP, lining progenitor cells; FLS, fibroblast-like synoviocytes; SubP, sublining progenitor cells; SubF, sublining fibroblasts; PSF, perturbed-state fibroblasts; Prolif, proliferating cells; OC, osteochondral lineage cells; Teno, tenocyte lineage cells; PreAd, pre-adipocytes; PeMy, perimysial cells.

**Supplementary Table 1. Single-cell RNA-seq datasets used in this study.**

| **GEO Accession** | **Injury model​** | **Injury type** | **Tissue dissection** | **Tissue disassociation** | **Cell sorting** | **Reference​** |
| --- | --- | --- | --- | --- | --- | --- |
| GSE214500​ | Joint surface injury (JSI)​ | Surgical scratch of the femoral cartilage | Knee joint | Collagenase | Gdf5-lineage and non-Gdf5-lineage fibroblasts by FACS | (1) |
| GSE231755​ | Destabilization of the medial meniscus (DMM)​​ | Surgical resection of the medial meniscotibial ligament | Synovium and fat pad | Collagenase and trypsin | CD45-/CD45+ cells by FACS | (2) |
| GSE211584​ | Anterior cruciate ligament rupture (ACLR)​ | Non-surgical excessive loading | Synovium and fat pad | Collagenase and liberase | none | (3) |

**Supplementary Table 2. Number of mice and cells obtained from datasets used in this study.**

| **Joint injury model​** | **Condition** | **Number of mice^1^** | **Pre-QC total cell number** | **Post-QC fibroblast number^2^** |
| --- | --- | --- | --- | --- |
| Joint surface injury (JSI)​  Surgical model​ | Control (naïve)​ | 2 | 1,274 | 1,108 |
|  | JSI 6 days | 4 | 3,696 | 2,875 |
| Destabilization of the medial meniscus (DMM)​  Surgical model​ | Control (naïve)​ | 20 | 8,997 | 2,953 |
|  | DMM 1 week | 8 | 13,974 | 5,064 |
|  | DMM 2 months | 12 | 10,754 | 4,973 |
| Anterior cruciate ligament rupture (ACLR)​  Non-surgical model​ | Control  (analgesia/anaesthesia only)​ | 4 | 3,035 | 1,012 |
|  | ACLR 7 days | 4 | 16,598 | 8,165 |
|  | ACLR 28 days | 4 | 8,113 | 4,189 |

^1^ In the DMM and ACLR studies, cells isolated from different mice were pooled prior to processing for scRNA-seq.

^2^ For QC, cells with fewer than 200 genes, more than 4,000 (control samples) or 6,000 genes (post-injury samples), or greater than 5% mitochondrial genes were excluded. In addition, cells negative for fibroblast markers *Pdpn* or *Pdgfra* and expressing either the hematopoietic cell markers *Ptprc* or *Fcer1g*, or the endothelial marker *Pecam1,* were computationally removed.

**Supplementary Table 3. Cellular composition of clusters**

| **Cluster** | **JSI Ctrl** | **JSI** | **DMM Ctrl** | **DMM 7d** | **DMM 2m** | **ACLR Ctrl** | **ACLR 7d** | **ACLR 28d** |
| --- | --- | --- | --- | --- | --- | --- | --- | --- |
| LinP | 15 | 197 | 181 | 162 | 282 | 2 | 205 | 43 |
| FLS | 29 | 566 | 329 | 306 | 1369 | 2 | 372 | 422 |
| SubP | 150 | 140 | 357 | 73 | 355 | 102 | 289 | 242 |
| SubF1 | 275 | 362 | 810 | 335 | 749 | 151 | 837 | 514 |
| SubF2 | 10 | 16 | 4 | 71 | 6 | 0 | 79 | 9 |
| PSF | 245 | 982 | 419 | 3182 | 1083 | 37 | 2792 | 664 |
| Prolif | 0 | 216 | 5 | 395 | 37 | 11 | 306 | 27 |
| OC | 43 | 87 | 1 | 50 | 30 | 0 | 299 | 40 |
| Teno | 61 | 53 | 70 | 62 | 197 | 398 | 1302 | 1280 |
| PreAd | 279 | 252 | 774 | 413 | 860 | 281 | 1067 | 738 |
| PeMy | 1 | 4 | 3 | 15 | 5 | 28 | 617 | 219 |

LinP, lining progenitor cells; FLS, fibroblast-like synoviocytes; SubP, sublining progenitor cells; SubF, sublining fibroblasts; PSF, perturbed-state fibroblasts; Prolif, proliferating cells; OC, osteochondral lineage cells; Teno, tenocyte lineage cells; PreAd, pre-adipocytes; PeMy, perimysial cells.

**References**

1. Collins FL, Roelofs AJ, Symons RA, Kania K, Campbell E, Collie-duguid ESR, et al. Taxonomy of fibroblasts and progenitors in the synovial joint at single-cell resolution. Ann Rheum Dis [Internet]. 2023 Mar 22;82(3):428–37. Available from: https://linkinghub.elsevier.com/retrieve/pii/S0003496724085157

2. Li J, Gui T, Yao L, Guo H, Lin YL, Lu J, et al. Synovium and infrapatellar fat pad share common mesenchymal progenitors and undergo coordinated changes in osteoarthritis. Journal of Bone and Mineral Research. 2024 Mar 22;

3. Knights AJ, Farrell EC, Ellis OM, Lammlin L, Junginger LM, Rzeczycki PM, et al. Synovial fibroblasts assume distinct functional identities and secrete R-spondin 2 in osteoarthritis. Ann Rheum Dis [Internet]. 2023 Feb;82(2):272–82. Available from: https://linkinghub.elsevier.com/retrieve/pii/S0003496724085868

4. Griss J, Viteri G, Sidiropoulos K, Nguyen V, Fabregat A, Hermjakob H. ReactomeGSA - Efficient Multi-Omics Comparative Pathway Analysis. Molecular and Cellular Proteomics. 2020 Dec 1;19(12):2115–24.

5. Subramanian A, Tamayo P, Mootha VK, Mukherjee S, Ebert BL, Gillette MA, et al. Gene set enrichment analysis: A knowledge-based approach for interpreting genome-wide expression profiles [Internet]. 2005. Available from: www.pnas.orgcgidoi10.1073pnas.0506580102

6. Liberzon A, Birger C, Thorvaldsdóttir H, Ghandi M, Mesirov JP, Tamayo P. The Molecular Signatures Database Hallmark Gene Set Collection. Cell Syst [Internet]. 2015 Dec 23;1(6):417–25. Available from: https://linkinghub.elsevier.com/retrieve/pii/S2405471215002185

7. Castanza AS, Recla JM, Eby D, Thorvaldsdóttir H, Bult CJ, Mesirov JP. Extending support for mouse data in the Molecular Signatures Database (MSigDB). Nat Methods [Internet]. 2023 Nov 13;20(11):1619–20. Available from: https://www.nature.com/articles/s41592-023-02014-7

8. Aibar S, González-Blas CB, Moerman T, Huynh-Thu VA, Imrichova H, Hulselmans G, et al. SCENIC: Single-cell regulatory network inference and clustering. Nat Methods. 2017 Oct 31;14(11):1083–6.

9. Cao J, Spielmann M, Qiu X, Huang X, Ibrahim DM, Hill AJ, et al. The single-cell transcriptional landscape of mammalian organogenesis. Nature [Internet]. 2019;566(7745):496–502. Available from: http://dx.doi.org/10.1038/s41586-019-0969-x

10. Zheng SC, Stein-O’Brien G, Augustin JJ, Slosberg J, Carosso GA, Winer B, et al. Universal prediction of cell-cycle position using transfer learning. Genome Biol [Internet]. 2022 Dec 31;23(1):41. Available from: https://genomebiology.biomedcentral.com/articles/10.1186/s13059-021-02581-y

11. Schwabe D, Formichetti S, Junker JP, Falcke M, Rajewsky N. The transcriptome dynamics of single cells during the cell cycle. Mol Syst Biol [Internet]. 2020 Nov 18;16(11). Available from: https://www.embopress.org/doi/10.15252/msb.20209946
